# Systematic Sonography Looking for Occult Wounds: accuracy of an abdominal ultrasound adjunct in penetrating trauma

**DOI:** 10.1186/s13089-020-00194-3

**Published:** 2020-11-26

**Authors:** Sri Devi Jagjit, Jordan Rupp, Robinson M. Ferre, Mary Kate Jordan, Brian Bales

**Affiliations:** 1 Georgetown Public Hospital Corporation, New Market St., Georgetown, Guyana; 2 Vanderbilt Medical Center, 1313 21st Avenue South, Oxford House 703, Nashville, TN 37212 USA; 3 Indiana University School of Medicine, Indianapolis, IN USA

## Abstract

**Background:**

Systematic Sonography Looking for Occult Wounds (SSLOW) in trauma is a novel technique for the evaluation of intra-abdominal wounds in penetrating trauma. No data exist regarding the effectiveness. The objective of this study was to evaluate the accuracy of the SSLOW exam.

**Methods:**

This is a prospective collected case series conducted over a period of 10 months and took place at the Accident and Emergency Department (A&E) of the Georgetown Public Hospital Corporation (GPHC). The study enrolled patients presenting to the A&E who were 16 years old or greater with penetrating abdominal trauma. All patients with penetrating trauma received an E-FAST examination. If the E-FAST examination was negative, a SSLOW examination was completed. The sonographer evaluated for free fluid collection between the loops of bowel. The results of the SSLOW were compared to usual care (surgery consult, serial abdominal and E-FAST exams, laparotomy, and 7-day follow-up) and then categorized into four groups: true positive, false positive, true negative, and false negative. These results lead to four categorical values. From these results, sensitivity, specificity, positive predictive value, negative predictive value, and likelihood ratios were calculated.

**Results:**

There were 5 (12%) true positives, 1 (2%) false positive, 37 (86%) true negatives, and zero (0%) false negative. The SSLOW was 100% sensitive (95% CI 5–100%) and 97% specificity (95% CI 74–96%). There was an 80% positive predictive value (95% CI 1.0–64% 95% CI) and 100% negative predictive value (95% CI 88–100%). The positive likelihood ratio was 8.4 (95% CI 3.69–19.1) and negative likelihood ratio was 0.

**Conclusion:**

The SSLOW examination may be a useful tool in the evaluation of penetrating abdominal injuries.

## Background

The Focused Assessment with Sonography for Trauma (FAST) is an established clinical tool utilized in the initial evaluation of trauma patients [1]. The FAST exam is done at the bedside and is applicable to both blunt and penetrating injuries [2]. It helps physicians identify intraperitoneal free fluid and free pericardial fluid [2]. The FAST has the advantage of being readily available, non-invasive, rapid, and repeatable; it does not involve any radiation and does not use contrast agents [2]. The FAST exam has further been expanded as the E-FAST to include the evaluation of the thoracic cavity for hemothoraces and pneumothoraces [3]. In a 1993 study of 476 patients by Rozycki et al. the sensitivity of the FAST in detecting fluid was found to be 79% with a specificity of 95.6% [4]. A prospective study performed by Ma and Mateer’s in 1995 showed that the E-FAST examination had a sensitivity of 90%, specificity of 99%, and an accuracy of 99% in detecting free intraperitoneal fluid and free pleural fluid in patients with both blunt and penetrating trauma [2]. A study done by Mckenney in 1996 showed that ultrasound has a sensitivity of 88%, specificity of 99%, and an accuracy of 97% for detecting intra-abdominal injuries in patients with suspected blunt abdominal trauma [5]. A more recent study published in 2017 also showed that the FAST examination has an excellent specificity of 98.4% [6].

Despite the improved ability of the FAST exam to recognize traumatic intra-abdominal injuries, small amounts of free fluid are difficult to detect with the traditional FAST exam. This limitation of the FAST exam is especially important in patients with penetrating abdominal injuries [4].

**Fig. 1:**
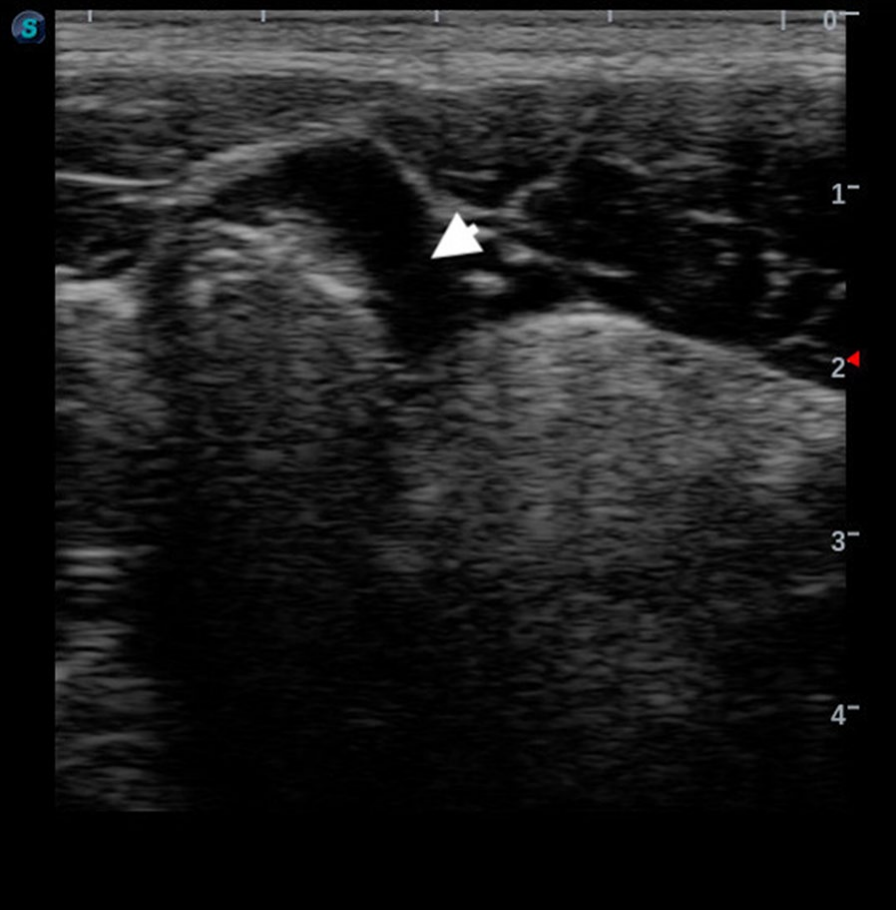
Positive SSLOW Exam. Positive SSLOW exam with free fluid in the bowel interloops. The patient had a penetrating wound to the right lower quadrant and was found to have an injury to the cecum on exploratory laparotomy

Occult penetrating wounds to the bowel are critical to identify. Delays in the identification of bowel injuries may result in the development of peritonitis, an infectious inflammation of the bowel lining associated with significant morbidity and mortality [7]. The Systematic Sonography Looking for Occult Wounds in Trauma (SSLOW) is a novel technique that may help augment sonographic findings of isolated bowel or solid organ injury from penetrating abdominal trauma [8]. A similarly described technique, the secondary FAST exam showed an increase in sensitivity of detecting free intraperitoneal fluid in blunt trauma from 70.7% for the primary (FAST) to 92.7% of the secondary exam [9]. The SSLOW exam is performed with the linear probe. The probe is placed in the right upper quadrant with the probe marker to the patient’s right and depth set so the peritoneal lining is central on the screen. The probe is dragged systematically moving up-and-down covering the entire area of the anterior abdomen. A SSLOW exam is considered positive when a pocket of free fluid large enough to form a geometric shape (e.g., triangle, trapezoid, etc.) is present between the loops of bowel (Fig. 1).

Georgetown Public Hospital Corporation (GPHC) is the only tertiary care referral hospital in the low resource South American country, Guyana. It is the principal teaching hospital of the University Of Guyana School Of Medicine. The Accident & Emergency Department cared for 44,000 patients in 2019 and health services are mostly free of cost. All major trauma patients in Guyana are referred to GPHC [10].

Trauma is a leading cause of morbidity and mortality worldwide as well as in Guyana and it is a top ten causes of death in low-income countries in 2016 [11, 12]. Upright chest X-ray identifies a small percentage of injuries [13]. Computed Tomography (CT) is expensive, not readily available, and has a limited sensitivity missing 9% of clinically significant bowel injuries [14]. Ultrasound is readily available in the Emergency Department at GPHC. The aim of this study was to determine the accuracy of the SSLOW exam compared to exploratory laparotomy, serial abdominal exams, further advanced imaging, or seven-day follow-up.

## Methods

The research was conducted over a period of 10 months, March 2018 to December 2018, and took place at the A&E of the GPHC. Patients were collected prospectively. The study size was determined by the duration of the primary investigator’s ultrasound fellowship. Inclusion criteria were patients presenting to the A&E who were 16 years old or greater with penetrating abdominal trauma. Sixteen is the legal age of consent in Guyana. Patients were enrolled when trained personnel were present. Exclusion criteria included abdominal evisceration, patients with known ascites, or a positive E-FAST exam. The specific aims were to determine the feasibility of utilizing the SSLOW examination and the accuracy among trauma patients at GPHC in comparison to the standard treatment: further advanced imaging, serial abdominal exams, and exploratory laparotomy. Patients were followed to discharge and with 7-day follow-up via phone call.

The SSLOW exams were performed by emergency medicine residents (in emergency residency training) and registrars (completed emergency medicine residency). The physicians working in the A&E receive structured ultrasound training as part of their emergency medicine residency program during which a minimum of 250 ultrasounds are performed and proctored [15]. Point-of-care ultrasound (POCUS) is used regularly in their practice. The SSLOW exam is an adaptation of basic skills used in other POCUS applications. For this study, each provider received a twenty-minute lecture on the SSLOW examination and two observed training scans with an ultrasound faculty. SSLOW examinations were supervised by a residency-trained emergency medicine specialist. The surgeons and surgical residents were informed of the SSLOW examination results. Approval for the study was granted from the GPHC and Vanderbilt University Medical Center Institutional Review Board (IRB).

All patients with penetrating trauma received full emergency medical stabilization per standard care, including an E-FAST. If the E-FAST examination was negative, a SSLOW examination was completed. The SSLOW examination was performed by an emergency medicine-trained physician or resident with additional ultrasound training and familiarity with the SSLOW technique. This study was performed with the patient in the supine position with a linear transducer: A Sonoscape S2 (SonoScape Medical Corporation, Shenzhen, China) with a 10 MHz linear probe. It was dragged across the entire abdomen in a systematic, lawn-mower fashion. The sonographer evaluated for free fluid collection between the loops of bowel with volume enough to form geometric shapes. The results of the SSLOW were compared to the standard. From these results, sensitivity, specificity, positive predictive value, negative predictive value, and likelihood ratios were calculated.

Patient results were recorded and communicated to the principal researcher. Patient consent was obtained by the treating physician. Data were collected on a data sheet by the treating physician and the form was handed over to the researcher. Follow-up of the patients were done by the researcher. Data were input into a Microsoft Excel (Microsoft Corp, Redmond, WA) sheet and were analyzed using Statistical Package for the Social Sciences (IBM, Armonk, NY) on a password protected computer.

## Results

A total of 43 patients were enrolled in this study. Any patient with a positive initial E-FAST examination was excluded. Patient demographics and details are included in Table 1. The mechanism of penetrating injury was mostly knife wounds at 44%, followed by wounds caused by ice picks 28% and scissors 19%. Four patients had other mechanisms of injury, one with a gunshot wound, one with a large piece of glass, another with a broken glass bottle, and the fourth with a scalpel. No injuries were self-inflicted.

**Table 1:** Demographics

Male	38 (88%)
Female	5 (12%)
Age groups	
16–20	16%
21–30	49%
31–40	19%
41–50	9%
51–60	7%

	Number (percentage)
Time of injury	
AM	15 (35%)
PM	28 (65%)
Time of presentation	
AM	12 (28%)
PM	31 (72%)
Mechanism of injury	
Knife	19 (44%)
Ice pick	12 (28%)
Scissors	8 (19%)
GSW	1 (2%)
Other	3 (7%)
Location of injuries	
RUQ	15 (35%)
RLQ	2 (5%)
LUQ	25 (58%)
LLQ	1 (2%)
Self-inflicted	
Yes	0
No	43 (100%)
Disposition	
Operating theatre	2 (5%)
Admission for observation	26 (60%)
Discharge	15 (35%)
Deceased	0

There were 6 positive SSLOW examinations. Among these 6 positives, one patient sustained a 3-cm stab wound to RLQ, had a negative FAST but a positive SSLOW, and was taken to the operating room for exploratory laparotomy. Exploratory laparotomy confirmed a 1-cm laceration to the cecum. The remaining five patients were managed expectantly. Four of these patients with initial negative FAST exams and positive initial SSLOW were later found to have positive FAST exams. These four patients were presumed to have minor solid organ injuries by the surgical team that did not require surgical exploration. There was one positive SSLOW examination with a negative E-FAST, who on repeated serial examinations and follow-up proved to have no otherwise confirmed injury. One patient with a negative FAST and a negative SSLOW was taken to the OR. The patient had a 3-cm stab wound with a knife to the LUQ, which violated the fascia. The patient had a negative exploratory laparotomy.

Of the total patients, 2 were taken to the OR and 26 patients were admitted for observations and were eventually discharged with a negative follow-up. They did not get readmitted or die. There were 15 patients who got discharged from the Emergency Department after a 6-h observation period, and their follow-ups were non-significant. All patients survived to 7-day follow-up.

The results are shown in Table 2. The SSLOW was 100% sensitive (95% CI 5–100%) and 97% specificity (95% CI 74–96%). There was an 80% positive predictive value (95% CI 1.0–64% 95% CI) and 100% negative predictive value (95% CI 88–100%). The positive likelihood ratio was 8.4 (95% CI 3.69–19.1) and negative likelihood ratio was 0.

**Table 2:** Categorical values

	True	False	
Positives	5	1	6
Negatives	37	0	37
	42	1	43

## Discussion

This is a small, preliminary study but the SSLOW exam proved to be useful for tool in the assessment of penetrating abdominal injuries. There were 6 positive SSLOW examinations, 5 were shown to have injuries on laparotomy or on other established imaging modalities. The evaluation of patients with abdominal injuries can be challenging, especially in countries with limited access to CT scans, such as Guyana. The early detection of occult penetrating wounds to the bowel is critical because a subtle bowel injury may require surgical exploration to prevent its progression. Complications, such as peritonitis, are associated with significant morbidity and mortality [7]. One previous study showed the use of a high frequency ultrasound after the traditional FAST improved the sensitivity of ultrasound to 97.2% in blunt trauma. No previous study has evaluated its use in penetrating injuries [9]. Victims of penetrating injuries are more likely to present upright or seated instead of in spinal precautions and more likely to have isolated bowel injuries. Free fluid may collect in different areas because of these differences.

In this small study, the SSLOW examination has shown to be a useful tool in detecting occult abdominal injuries. Thus, the SSLOW examination may be an adjunct that will help surgeons in early detection of isolated bowel or solid organ injuries.

## Limitations

Due to the small sample size, the full examination of the SSLOW exam test characteristics is a work in progress. Additionally, there were few positive SSLOW exams. Future studies are needed with a large number of confirmed intra-abdominal injuries. The SSLOW examinations were done by trained ultrasound personnel and images were reviewed by an ultrasound-trained fellow. This might not be applicable to a population with no formal ultrasound training.

## Conclusion

The SSLOW examination may be a useful tool in the evaluation of penetrating abdominal injuries at the Emergency Department of the Georgetown Public Hospital Corporation. This modality proved to be very sensitive and specific in detecting isolated bowel injuries and solid organ injuries in patients with penetrating abdominal trauma as an adjunct to the current standard of care.

## Data Availability

Original data will be made available upon reasonable request to the corresponding author.
